# Association of Toll-like receptor 4 polymorphism with age-dependent systolic blood pressure increase in patients with coronary artery disease

**DOI:** 10.1186/s12979-015-0031-2

**Published:** 2015-05-20

**Authors:** Simon Schneider, Werner Koch, Petra Hoppmann, Romy Ubrich, Stephan Kemmner, Eva Steinlechner, Uwe Heemann, Karl-Ludwig Laugwitz, Adnan Kastrati, Marcus Baumann

**Affiliations:** Medizinische Klinik und Poliklinik, Klinikum rechts der Isar, Technische Universität München, Ismaninger Str.22, 81675 Munich, Germany; Department of Nephrology, Klinikum rechts der Isar, Technische Universität München, Ismaninger Str.22, 81675 Munich, Germany; Deutsches Herzzentrum München, Technische Universität München, Lazarettstr. 36, 80636 Munich, Germany; German Centre for Cardiovascular Research (DZHK), partner site Munich Heart Alliance, Oudenarder Straße 16, 13347 Munich, Germany

**Keywords:** Hypertension, Ageing, Toll-like receptor 4, TLR4

## Abstract

**Background:**

Systolic blood pressure (SBP) increases steadily with age and bears an independent continuous relationship with the incidence of cardiovascular events. Low-grade inflammation is a suspected pathomechanism causing vascular aging and promote coronary artery disease (CAD). Recent animal studies give evidence that Toll-like receptor 4 (TLR4) modulate inflammation and contribute to age-dependent SBP increase. However, there are no data about TLR4 and age-dependent blood pressure increase in human.

**Methods and results:**

We therefor investigate a human cohort of 2679 patients with CAD aged between 50–80 years. Genotypes were determined for the TLR4 single nucleotide polymorphism rs4986790 (TLR4 896A/G). Patients were stratified according to tertiles of age and the upper tertile was compared to lower tertiles.

In this cohort we show that older patients with the TLR4 896 G allele had significantly lower SBP (TLR4 G allele carriers: 148.2 ± 30.4 mmHg versus A/A allele carrier: 154.9 ± 27.2 mmHg; *P* < 0.05) and lower pulse pressure (TLR4 G allele carriers: 69.1 ± 29.7 mmHg versus A/A allele carrier: 75.5 ± 26.4 mmHg; *P* < 0.05) as compared to TLR4 896A/A allele carrier.

**Conclusion:**

We demonstrate an association between the TLR4 SNP rs4986790 genotype and age-dependant blood pressure increase in patients with coronary artery disease.

**Electronic supplementary material:**

The online version of this article (doi:10.1186/s12979-015-0031-2) contains supplementary material, which is available to authorized users.

## Background

Systolic blood pressure (SBP) increases steadily with age and bears an independent continuous relationship with the incidence of cardiovascular events [1]. More than 50 % of people aged 60+ are hypertensive and the majority has isolated systolic hypertension [2]. Among cardiovascular risk factors amenable to prevention in the elderly, systolic hypertension is of major importance [3]. Several interventional trials demonstrated the clinical relevance of lowering SBP in elderly individuals for reducing cardiovascular events and improving survival [4, 5]. Therefore, understanding the pathophysiology of SBP increase with lifetime is of major interest for future therapeutic interventions.

Chronic low-grade inflammation plays a crucial role in hypertension during aging [6–9]. This role has been poorly explored, although chronic low-grade inflammation has been suggested as an important aspect of aging as well as coronary artery disease (CAD) [10]. The Toll-like receptor (TLR) 4 is an evolutionarily ancient pattern recognition receptor that plays a key role in the innate immune response [11]. In this context it reflects a major regulator of inflammation linked to endogenous and exogenous ligands [12]. Endogenous TLR4 ligands are present in physiological processes of apoptosis or necroptosis throughout lifetime [13, 14]. Exogenous TLR4 ligands like fragments of gram-negative bacterials are exposed in faeces and the intestinal tract [15]. Together, these ligands induce TLR4 signaling and participate in inflammation.

The role of TLR4 polymorphism in CAD and myocardial infarktion was inconsistant in the literature [16, 17]. Recent investigations on circulating Toll-like receptor 4 (TLR4)-responsive miRNA expression shows elevated levesl of TLR4 protein in patients with CAD [18]. Animal data gives first evidence that TLR4 signaling might contribute to chronic low-grade inflammation, which influence SBP increase [19] and hypertension-associated cardiac remodeling [20]. The common missense mutation Asp299Gly (rs 4986790) affects the extracellular domain of the TLR4 receptor and is associated with a blunted response to TLR4 ligands like lipopolysaccharide (LPS) in humans [21]. Therefore, we investigated age associated blood pressure increase in a large clinical cohort of patient with CAD or myocardial infarction in relation the functional TLR4 single nucleotide polymorphism rs 4986790.

## Results

We investigated 2679 patients with myocardial infarction stratified by age and genotype of the functional TLR4 SNP rs4986790 (Asp299Gly) [16]. Dividing the cohort according to age by tertiles, the genotype distribution of the polymorphism rs4986790 was not significantly different between the age related tertiles. TLR4 896G allele carriers of the upper age tertile (age ≥70 and <80 years) had a 7 mmHg significantly lower SBP compared to homozygot AA allele carriers (TLR4 G allele carriers: 148.2 ± 30.4 mmHg versus A/A allele cariers: 154.9 ± 27.2 mmHg; *P* < 0.05). This is also correlated to a reduced number of hypertensive patients in this group (Table 1). Pulse pressure as marker for aortic damage was reduced by six mmHg (*p* < 0.05). Univariate analysis confirmed age (*p* < 0.001) and sex (*p* < 0.001) as commonly known blood pressure modulating risk factors in all tertiles of the cohort. Interestingly TLR4 G polymorphism shows a significant association for SBP (*p* = 0.017) (Table 2), hypertension (*p* < 0.001) and pulse pressure (*p* = 0.026) only in the upper age tertile (age ≥70 and <80 years) (Additional file 1: Table S1). These effects were not present in the lower age tertiles (age ≥50 and <70) (*p* = 0.56). Multivariate analysis of the upper age tertile confirm TLR4 896 G polymorphism as independent predictor of SBP and hypertension (Table 3)

**Table 1:** Comparison of TLR4 896 A/A and TLR4 896 AG/GG Genotype divided by Age Tertiles

Characteristics	Total cohort (*n* = 2679)		Lower and middle age tertiles age ≥50 and <70 (*n* = 1772)		Upper age tertile age ≥70 and <80 (*n* = 907)	
Genotype	AA	AG/GG	AA	AG/GG	AA	AG/GG
Patients, n (%)	2393 (89.3)	286 (10.7)	1590 (89.7)	182 (10.3)	803 (88.6)	104 (11.4)
Age, y	65.5 ± 8.0	65.7 ± 8.0	61 ± 5.4	60.9 ± 5.5	74.6 ± 2.8^**#**^	74.4 ± 2.6^**#**^
Sex, female %	24	25	20	21	33^**#**^	33^**#**^
BMI, kg/m2	27.0 ± 3.8	27.0 ± 4.0	27.3 ± 3.8	27.5 ± 4.1	26.5 ± 3.8	26.2 ± 3.6
SBP, mmHg	150.6 ± 26.8	148.6 ± 28.2	148.2 ± 26.3	148.9 ± 26.9	154.9 ± 27.2^**#**^	148.2 ± 30.4*
DBP, mmHg	79.4 ± 4.3	79.0 ± 4.8	79.7 ± 4.2	79.2 ± 4.5	79.3 ± 4.6	78.6 ± 5.4
PP, mmHg	71.2 ± 26.3	68.8 ± 28.0	69 ± 25.9	68,5 ± 28.0	75,5 ± 26.4^**#**^	69,1 ± 29.7*
HR, bpm	73.8 ± 12.3	73.3 ± 11.7	73.4 ± 12.0	73.6 ± 11.8	74.5 ± 12.8	72.7 ± 11.5
Hypertension, %	69	64	66	66	76^**#**^	60*
Hypercholesterolemia, %	57	57	58	61	55	50
Diabetes, %	22	24	19	24	27^**#**^	24
Smoking, %	47	49	54	54	34^**#**^	40^**#**^

AA versus AG/GG *P < 0.05, Lower age tertiles versus higher age tertile ^#^ *P* < 0.05

**Table 2:** Univariate analysis for systolic blood pressure of the whole cohort and devided by tertiles

A: Whole cohort Parameter				
	Coefficient B	SEM	Beta	*P*
Age, y	0.509	0.064	0.151	<0.001
Sex (M = 0,F = 1)	10.943	1.197	0.174	<0.001
BMI, kg/m^2^	0.05	0.138	0.007	0.7
Diabetes (No = 0, Yes = 1)	3.445	1.256	0.053	0.006
Hypercholesterolemia (No = 0, Yes = 1)	2.987	1.053	0.055	0.005
TLR4 SNP 896 (AA = 0, AG/GG = 1)	−1.595	1.712	−0.018	0.35
B: Lower and middle age tertiles (age ≥50 and <70)				
Parameter	Coefficient B	SEM	Beta	*P*
Age, y	0.719	0.114	0.149	<0.001
Sex (M = 0,F = 1)	11.624	1.550	0.176	<0.001
BMI, kg/m^2^	0.274	0.166	0.04	0.1
Diabetes (No = 0, Yes = 1)	5.755	1.577	0.087	<0.001
Hypercholesterolemia (No = 0, Yes = 1)	2.416	1.273	0.045	0.058
TLR4 SNP 896 (AA = 0, AG = 1)	1.211	2.092	0.014	0.56
C: Upper age tertile (age ≥70 and <80)				
Parameter	Coefficient B	SEM	Beta	*P*
Age, y	0.492	0.324	0.051	0.13
Sex (M = 0,F = 1)	8.324	1.938	0.142	<0.001
BMI, kg/m^2^	−0.148	0.246	−0.021	0.54
Diabetes (No = 0, Yes = 1)	−1509	2.075	−0.024	0.46
Hypercholesterolemia (No = 0, Yes = 1)	4.716	1.845	0.085	0.011
TLR4 SNP 896 (AA = 0, AG/GG = 1)	−7.023	2.931	−0.08	0.017

**Table 3:** Multivariate analysis for systolic blood pressure and hypertension of upper age tertile (age ≥70 and <80)

A: Dependent variable: Systolic blood pressure				
Parameter	Coefficient B	SEM	Beta	*P*
Age, y	0.351	0.321	0.036	0.27
Sex (M = 0,F = 1)	7.667	1.951	0.13	<0.001
Hypercholesterolemia (No = 0, Yes = 1)	3.797	1.838	0.068	0.039
TLR4 SNP 896 (AA = 0, AG/GG = 1)	−6.682	2.901	−0.076	0.022
B: Dependent variable: Hypertension				
Parameter	Coefficient B	SEM	Beta	*P*
Age, y	0.007	0.005	0.047	0.16
Sex (M = 0,F = 1)	0.094	0.031	0.1	0.003
Hypercholesterolemia (No = 0, Yes = 1)	0.056	0.029	0.063	0.057
TLR4 SNP 896 (AA = 0, AG/GG = 1)	−0.160	0.046	−0.114	0.001

This analysis demonstrates in humans that 896G allele carriers older than 70 years reveal lower SBP suggesting an effect of TLR4 signaling on SBP increase with aging.

## Discussion

The increasing incidence of hypertension with age is thought to be a physiological process which develops with vascular aging mediated by low-grade inflammation and oxidative stress. Animal studies demonstrated that oxidative stress is acutely induced in the aortic tissue by Toll like receptor 4 (TLR4) ligands. TLR4 are present in different vascular beds and TLR4 ligands have been shown to induce chronic low-grade inflammation and oxidative stress in vascular smooth muscle cells [22, 23]. Even more, endogenous TLR4 ligands are strongly involved in a mouse model of acute blood pressure increase [19].

We investigated the human TLR4 SNP rs4986790 (Asp299Gly; 896 A/G) in 2679 patients with history of myocardial infarction [16]. This polymorphism affects the extracellular domain of the TLR4 receptor and is associated with a blunted response to inflammatory ligands in humans [21]. We could show that carriers of the TLR4 896 G genotype presented a significant lower SBP starting from the age of 70 years. Other studies investigating genetic variants which influence on blood pressure did neither detect an association with TLR4 [24] nor the SNP rs4986790 [25]. This is explained by the relatively low mean age of the cohorts with 52.5 ± 10.1 years compared to our cohort with a mean age of 65.7 ± 8.0.years. Correspondingly a large epidemiological study in young healthy Finnish subjects showed no association with Toll-like receptor 4 gene (Asp299Gly) polymorphism and blood pressure [26]. In this cohort the mean age was below 40. At this age no significant SBP increase due to aging is suspected. Other smaller human cohorts in patients aged 60 had only mild non-significant blood pressure differences [27, 28]. Another explanation might be the stratification of the cohort by age in tertiles. Also, we found no difference in blood pressure when examining the entire cohort without consideration of age. But comparison of upper and lower age tertiles identified a significant association of the TLR4 SNP rs4986790 with SBP, pulse presser and hypertension. However, no other study included such a large group of aged patients 70+. Additionally CAD composes a high-risk population with particular chronic low-grade inflammation which may have contributed to the blood pressure effect.

Vascular aging is accompanied by vascular damage. Pulse pressure is an accepted parameter of vascular damage. Our data confirm that patients without the functional TLR4 SNP have a pulse pressure increase with age corresponding to the general human population [29, 30]. By contrast, the patients containing the TLR4 loss-of-function SNP rs4986790 had no increase in pulse pressure. This association suggests that increase in pulse pressure with aging might be affected by TLR4 and ameliorated by the TLR4 loss-of-function SNP.

The probability of a false negative association result because of population stratification is relatively low, because the study participants were consecutively recruited from a defined geographic area of southern Germany with limited recent immigration. The sample size allowed an analysis with 80 % power to detect a six mmHg decrease of SBP among the carriers of the 896G allele at a two-sided **a**-error of 0.05.

In summary, our data indicate for this large cohort of patients with myocardial infarction that carriers of the TLR4 SNP rs4986790 starting from the age of 70 years have a lower SBP and pulse pressure. We propose that TLR4 signaling influences age-dependent SBP increase in CAD patients.

## Conclusion

We demonstrate an association between the TLR4 SNP rs4986790 genotype and age-dependant blood pressure increase in large cohort of patients with coronary artery disease.

## Methods

### Patients

Participants were examined at 1. Medizinische Klinik rechts der Isar, Technischen Universität München or Deutsches Herzzentrum München. Between 1993 and 2002, patients with myocardial infarction between the ages of 50 to 80 years were enrolled in a dedicated registry. Written informed consent for genetic analysis was obtained from all individuals. In no case, consent was withdrawn. Out of this cohort, patients with cardiogenic shock and advanced heart insufficiency defined as a SBP < 100 mmHg were excluded, finally reaching a cohort size of 2679 individuals. SBP and DBP were determined under standardized conditions in the laying position (Cardis, Schwarzer GmbH). Pulse pressure was calculated from systolic and diastolic blood pressure. Clinical and laboratory data were prospectively collected in an online database. Source data check was done with 20 % of the data. The Institutional Ethics Committee approved the study protocol and the reported investigations were in accordance with the principles of the current version of the Declaration of Helsinki.

### Definitions

The diagnosis of myocardial infarction was defined according to the European Society of Cardiology. Briefly, patients presenting with chest pain lasting >20 min combined with ST-segment elevation or pathological Q waves on a surface electrocardiogram. Patients with myocardial infarction had to show either an angiographically occluded infarct-related artery or regional wall motion abnormalities corresponding to the electrocardiographic infarct localization or both. Systemic arterial hypertension was defined as a systolic blood pressure of ≥140 mmHg and/or a diastolic blood pressure of ≥90 mmHg, on at least two separate occasions, or anti-hypertensive treatment. Hypercholesterolemia was defined as a documented total cholesterol value ≥240 mg/dL (≥6.2 mmol/L) or current treatment with cholesterol-lowering medication. Persons reporting regular smoking in the previous 6 months were considered as current smokers. Diabetes mellitus was defined as the presence of a fasting glycaemia ≥126 mg/dL or patients on antidiabetic treatment.

### Determination of *TLR4* genotype

A blood sample was drawn from each participant during the regular routing examination for coronary artery angiogram. Genomic DNA was extracted from peripheral blood leukocytes with the QIAamp DNA Blood Kit (Qiagen, Hilden, Germany) or the High Pure PCR Template Preparation Kit (Roche Applied Science, Mannheim, Germany). Genotype analysis was performed with allele-specific fluorogenic oligonucleotide probes in an assay combining the polymerase chain reaction (PCR) and the 5′ nuclease reaction (TaqMan technique; Applied Biosystems, Darmstadt, Germany). Primers and probes were established with the Primer Express software (version 2.0.0; Applied Biosystems) after import of a DNA sequence (Homo sapiens *TLR4* gene, TLR4A allele) deposited under accession number AF177765 in GenBank (http://www.ncbi.nlm.nih/entrez/). The primers used for amplification of the 896A/G SNP (Asp299Gly; rs4986790) were 5′ TTGAAGAATTCCGATTAGCATACTTAGAC 3′ and 5′ TCACCAGGGAAAATGAAGAAACAT 3′. Allele-specific signalling was accomplished with the use of the fluorogenic dyes 6-carboxy-fluorescein (FAM) and VIC (proprietary dye of Applied Biosystems) which were attached to the 5′ end of the probe oligonucleotides. Minor groove binder (MGB) groups were conjugated with the 3′ end of the oligonucleotides to facilitate formation of stable duplexes between the probes and their single-stranded DNA targets. The structures of the probes were as follows (allele-specific nucleotides are underlined): FAM-5′ CCTCGATGATATTATTGACT 3′-MGB (for 896A), VIC-5′ CTCGATGGTATTATTG 3′-MGB (for 896G) [31]. Oligonucleotides were synthesized by Applied Biosystems. The two-step thermocycling procedure consisted of 35 cycles of denaturation at 92 °C for 15 s and primer annealing and extension at 60 °C for 1 min. After cycling on a GeneAmp PCR System 9600 or 9700 (Applied Biosystems), endpoint fluorescent data acquisition and genotype calling was achieved on an ABI Prism 7000 Sequence Detection System (Applied Biosystems). As a control, genotyping was repeated for 20 % of the samples with the use of DNA prepared separately from the original blood sample. The ability of the TaqMan systems to provide correct genotype data was verified in separate analyses of 200 different PCR products with the allele-discriminating restriction enzyme *Bcc*I (New England Biolabs, Frankfurt am Main, Germany) (896A/G SNP). The results obtained with the different methods were fully corresponding, which demonstrated the reliability of the TaqMan systems that were used for genotyping of the 896A/G SNPs in the entire study population. Genotype determination was done by workers who had no knowledge of clinical, laboratory, or angiographic data of the individuals enrolled in the study.

### Statistical analysis

Patients in the cohort were divided by age into tertiles. In order to investigate the age-related effect, the lower two tertiles were summarized (age ≥50 and <70) and compared with the upper tertile (age ≥70 and <80). The association was examined by the univariate analysis using Spearman’s correlation test. The analysis consisted of comparing separate genotype distributions, between the age tertiles of patients with MI. Association analyses for SBP and DBP were conducted using linear regression, and analyses for dichotomous hypertension using logistic regression Discrete variables are expressed as counts (percentage) and compared with the use of the *χ*^2^ test or Fisher's exact test, as appropriate. Continuous variables are expressed as mean ± SD and compared by means of the unpaired, two-sided *t* test. Genotype frequencies in the case group were compared with values predicted by Hardy–Weinberg equilibrium with the use of the *χ*^2^ test. Univariate analysis was performed for the whole cohort, upper age tertile and both lower age tertiles. Multivariate analysis used age, gender and hypercholesterolemia. Level of significance was set at *p* < 0.05.

## Additional file

Additional file 1: Table S1. Univariate analysis.
