# Supplementary material for: Association of Toll-like receptor 4 polymorphism with age-dependent systolic blood pressure increase in patients with coronary artery disease
Source: Immun Ageing. 2015 May 20;12:4. doi: 10.1186/s12979-015-0031-2 (PMC4443624; doi:10.1186/s12979-015-0031-2)
Supplement: Additional file 1: Table S1. — Univariate analysis. [file 12979_2015_31_MOESM1_ESM.docx]

**Supplemental Table 1. Univariate analysis**

1. Dependent variable: Hypertension

**Whole cohort**

| **Parameter** | **Coefficient B** | **SEM** | **Beta** | ***P*** |
| --- | --- | --- | --- | --- |
| Age, y | 0.007 | 0.001 | 0.114 | <0.001 |
| Sex (M=0,F=1) | 0.126 | 0.021 | 0.117 | <0.001 |
| BMI, kg/m^2^ | 0.004 | 0.002 | 0.032 | 0.104 |
| Diabetes (No=0, Yes=1) | 0.027 | 0.022 | 0.024 | 0.22 |
| Hypercholesterolemia (No=0, Yes=1) | 0.038 | 0.018 | 0.041 | 0.034 |
| TLR4 SNP 896 (AA=0, AG=0) | -0.049 | 0.029 | -0.032 | 0.10 |

**Lower and middle age tertiles (age ≥50 and <70)**

| **Parameter** | **Coefficient B** | **SEM** | **Beta** | ***P*** |
| --- | --- | --- | --- | --- |
| Age, y | 0.009 | 0.002 | 0.104 | <0.001 |
| Sex (M=0,F=1) | 0.126 | 0.028 | 0.106 | <0.001 |
| BMI, kg/m^2^ | 0.007 | 0.003 | 0.055 | 0.024 |
| Diabetes (No=0, Yes=1) | 0.060 | 0.028 | 0.051 | 0.034 |
| Hypercholesterolemia (No=0, Yes=1) | 0.027 | 0.023 | 0.028 | 0.24 |
| TLR4 SNP 896 (AA=0, AG=1) | 0.014 | 0.037 | 0.009 | 0.71 |

**Upper age tertile (age ≥70 and <80)**

| **Parameter** | **Coefficient B** | **SEM** | **Beta** | ***P*** |
| --- | --- | --- | --- | --- |
| Age, y | 0.009 | 0.005 | 0.059 | 0.075 |
| Sex (M=0,F=1) | 0.105 | 0.031 | 0.112 | <0.001 |
| BMI, kg/m^2^ | 0.001 | 0.004 | 0.012 | 0.73 |
| Diabetes (No=0, Yes=1) | -0.043 | 0.033 | -0.044 | 0.19 |
| Hypercholesterolemia (No=0, Yes=1) | 0.068 | 0.029 | 0.078 | 0.02 |
| TLR4 SNP 896 (AA=0, AG=1) | -0.166 | 0.046 | -0.118 | <0.001 |

1. **Dependent variable: Pulse pressure**

**Whole cohort**

| **Parameter** | **Coefficient B** | **SEM** | **Beta** | ***P*** |
| --- | --- | --- | --- | --- |
| Age, y | 0.020 | 0.003 | 0.147 | <0.001 |
| Sex (M=0,F=1) | 0.007 | 0.062 | 0.003 | 0.90 |
| BMI, kg/m^2^ | 0.016 | 0.007 | 0.058 | 0.019 |
| Diabetes (No=0, Yes=1) | 0.308 | 0.063 | 0.119 | <0.001 |
| Hypercholesterolemia (No=0, Yes=1) | -0.182 | 0.054 | -0.082 | 0.001 |
| TLR4 SNP 896 (AA=0, AG=1) | -0.108 | 0.087 | -0.030 | 0.22 |

**Lower and middle age tertiles (age ≥50 and <70)**

| **Parameter** | **Coefficient B** | **SEM** | **Beta** | ***P*** |
| --- | --- | --- | --- | --- |
| Age, y | 0.024 | 0.006 | 0.121 | <0.001 |
| Sex (M=0,F=1) | -0.060 | 0.081 | -0.022 | 0.46 |
| BMI, kg/m^2^ | 0.031 | 0.008 | 0.112 | <0.001 |
| Diabetes (No=0, Yes=1) | 0.307 | 0.079 | 0.116 | <0.001 |
| Hypercholesterolemia (No=0, Yes=1) | -0.150 | 0.066 | -0.068 | 0.023 |
| TLR4 SNP 896 (AA=0, AG=1) | -0.109 | 0.108 | -0.030 | 0.32 |

**Upper age tertile (age ≥70 and <80)**

| **Parameter** | **Coefficient B** | **SEM** | **Beta** | ***P*** |
| --- | --- | --- | --- | --- |
| Age, y | 0.741 | 0.334 | 0.077 | 0.027 |
| Sex (M=0,F=1) | 8.218 | 1.985 | 0.142 | <0.001 |
| BMI, kg/m^2^ | 0.154- | 0.247 | 0.022- | 0.54 |
| Diabetes (No=0, Yes=1) | -1.652 | 2.123 | -0.027 | 0.43 |
| Hypercholesterolemia (No=0, Yes=1) | 4.826 | 1.882 | 0.089 | 0.011 |
| TLR4 SNP 896 (AA=0, AG=1) | -6.613 | 2.961 | -0.077 | 0.026 |
